# Prevalence of low back pain and disability among secondary school teacher in the eastern province of the Kingdom of Saudi Arabia: a cross-sectional analytical study

**DOI:** 10.3389/fpubh.2024.1307845

**Published:** 2024-01-12

**Authors:** Abdulelah H. Almansour, Danah S. Almutairi, Turki M. Alaskar, Mohannad S. Kalkatawi, Mohammed F. Aljubair, Rakan S. Alotaibi, Khalid S. AlHarkan, Hatem A. Alqahtani, Reem S. AlOmar

**Affiliations:** ^1^Department of Family and Community Medicine, College of Medicine, Imam Abdulrahman Bin Faisal University, Dammam, Saudi Arabia; ^2^College of Medicine, Imam Abdulrahman Bin Faisal University, Dammam, Saudi Arabia; ^3^Department of Occupational Medicine, Khobar Health Network, Eastern Health Cluster, Ministry of Health, Khobar, Saudi Arabia; ^4^Department of Public Health, Health Sciences College Al-Leith, Umm Al-Qura University, Mecca, Saudi Arabia; ^5^Erada Complex and Mental Health, Eastern Health Cluster, Ministry of Health, Dammam, Saudi Arabia; ^6^Department of Occupational Medicine, Ministry of Defense, Khobar, Saudi Arabia

**Keywords:** low back pain, disability, teachers, work related, occupational health, epidemiology

## Abstract

**Introduction:**

Lower back pain is common worldwide and affects over 600,000 people annually, including teachers. The study aimed to investigate the prevalence of low back pain and disability among secondary school teachers in the Eastern Province of the Kingdom of Saudi Arabia.

**Materials and methods:**

This cross-sectional study included secondary school teachers in the eastern province of Saudi Arabia. 34 schools were selected using a multistage stratified sampling approach. Teachers were allotted randomly and proportionally to each school. Data was collected by anonymous questionnaire having three elements: sociodemographic and health-related questions, the Standardized Nordic Questionnaire, and the Oswestry Low Back Pain Disability Questionnaire. The anthropometric data was also included. Both unadjusted and adjusted logistic regression analyses were performed.

**Results:**

A total of 601 teachers participated in the study with 62.56% reported low back pain. The overall mean age was 40.31 ± 8.13 years. The male-to-female ratio was similar. Back pain was significantly higher among females than males (73.36 and 51.52%, respectively). Additionally, back pain will significantly increase when stress levels and the number of classes increases. A positive correlation was found between age with low back pain (*p* = 0.001).There was minimal disability in 64.63% of the 376 teachers who reported low back pain, moderate disability in 29.79%, and severe disability in 4.79%, and only three (0.8%) were considered crippled. Females were more frequently seen in moderate and crippled categories, and perceived stress levels generally increased mean disability scores. Age and female gender were revealed to be significant predictors of low back pain by logistic regression (adjusted odds ratio [OR] = 1.04, 95% confidence interval [CI] = 1.02–1.07) and (adjusted OR = 2.11, 95% CI = 1.45–3.05), respectively. The number of classes per week was also a significant predictor.

**Conclusion:**

This study adds to the epidemiological evidence that reveals a high prevalence of low back pain and disability among teachers. Identified risk factors in this study may also reinforce the importance of setting different interventions and preventive measures to reduce lower back pain risk.

## 1 Introduction

Lower back pain (LBP) is characterized as pain or discomfort between the costal margins and the inferior gluteal folds, with or without leg pain and it may arise from a single traumatic event or it may develop gradually over time as a result of microtrauma caused by repetitive activity. LBP is one of the most frequently reported symptoms in primary care settings worldwide. Most patients seek medical attention for lower back pain because of the obvious limitations it imposes on their daily lives and how it significantly impairs their overall occupational performance. Furthermore, low back pain is one of the most prevalent medical conditions reported in working individuals. One study indicated that up to 80% of individuals experienced back pain at some point in their life (1, 2).

According to the National Institute for Occupational Safety and Health (NIOSH), back disorders affect more than 600,000 employees each year in the United States of America (USA), costing approximately $50 billion annually. Back injuries and disorders are expected to increase in frequency and economic impact on the workforce over the next several decades as the average lifespan of the workforce increases and healthcare costs rise (1).

A study published in 2020 aimed at estimating the burden of LBP globally between the years 1990–2017 concluded that there was a rising trend in years lived with disability (YLDs) attributed to LBP during that time, stating that the global YLDs back in 1990 was 42.5 million, which increased to 64.9 million by 52.7% in 2017 (3).

Teachers are more prone to this condition and its effects. Many studies have shown that a substantial percentage of teachers suffer from low back pain (4–8). Poor posture, prolonged sitting while working on students’ homework and preparing for lessons are some of the factors that contribute to low back discomfort in teachers. LBP results in poorer life quality, higher absenteeism, lower labor productivity, and early retirement. Furthermore, LBP has a significant financial impact on the healthcare system (7, 9).

A study conducted in Chile in the year 2021 concluded that there indeed was a strong association between teachers’ reporting a lower quality of life and having musculoskeletal pains or disorders acquired through teaching or at the workplace. Two additional studies conducted in 2022 found that female teachers were more prone to this pain-induced reduction in the quality of life (10–12).

Over the last 10 years, several studies have been conducted on the prevalence of LBP among teachers, both globally and in Saudi Arabia. In 2013, Darwish et al. conducted a study in Saudi Arabia to examine the prevalence of Musculoskeletal Pain Disorders among female Saudi secondary school teachers. In Darwish et al.’s study, the prevalence of musculoskeletal pain disorders was 79.17%. The main point of pain was the lower back (63.8%). Another cross-sectional study conducted among secondary school teachers in Hail, Saudi Arabia found that the prevalence of low back pain was 62.55%, making it the most frequent musculoskeletal disorder (13, 14).

A comprehensive review, which included 11 relevant studies with a total of 5,805 schoolteachers to evaluate the pooled prevalence and related variables of low back pain among teachers in Africa, revealed a high prevalence of back pain, indicated to be 59.0% (15).

Up to now, a number of studies have examined the association between different variables and low back pain. The relationship between engaging in physical activity and musculoskeletal disorders has been investigated in Grabara study. The author also noticed that teachers with more musculoskeletal disorders—including back pain—were less likely to participate in both vigorous and overall physical activity than those with fewer painful body parts. The lower back was also shown to be the most frequently affected area and to have the highest average pain intensity (16).

Another risk factor for musculoskeletal disorder -including LBP- among teaching professionals that was proposed by a study published in 2018 is the level of perceived stress, as the study focused on the mind–body connection concluding that occupational stress could in fact lead to the development of physical symptoms, such as low back pain (17).

Some work-related factors such as the number of hours taught while standing, the duration of sitting teaching, working on a head-down posture, and chairs with inadequate back support can increase the odds of developing musculoskeletal disorders including back pain as found in study conducted on teachers in Machakos County, Kenya (18).

A study by Bontrup et al. (19) examined the relationship between sedentary office workers’ sitting behavior and low back pain. He noticed a stronger correlation between sitting behavior and chronic low back pain (LBP) than for with acute pain or disability. This finding may be explained by the fact that people with chronic LBP are more aware of pain-free sitting positions and pain-provoking movements than people with acute pain.

To our knowledge, few recent studies have been conducted on our population of interest to study the prevalence of LBP, its related disabilities and related risk factors. Therefore, the current study aimed to investigate the prevalence and level of disability of low back pain among secondary school teachers of both genders in the eastern region of Saudi Arabia, considering several lifestyle and work-related factors as well as a number of demographic variables.

## 2 Methodology

### 2.1 Study design and participants

This cross-sectional study was conducted between January and June 2022. It included teachers from both government and private secondary schools in the Eastern Province of the KSA. The inclusion criteria were male and female teachers aged 22–62 years. Non-Saudis and those with nonteaching responsibilities, such as administrators, were excluded. Participants with histories of back injury, surgery, or inflammation were excluded.

According to the Ministry of Education (MoE), the total number of teachers in secondary schools in the Eastern province was 5,752. Using this number as the total population and the prevalence of 57.3% of the estimated LBP among teachers (20), with a precision of 4% and an alpha level of 0.05, the estimated minimum required sample size was 533. The Eastern Province is home to three large districts: Dammam, Khobar, and Dhahran. Seventeen schools from Dammam and another 17 from both Khobar and Dhahran were chosen using a multistage stratified sampling technique. The total number of teachers was determined through a proportional allocation. Once the school and the number of teachers within that school were determined, a list of all teachers was created and numbered from 1 to n. A simple random sampling technique was used to select teachers for participation.

This study was approved by the Institutional Review Board of the Imam Abdulrahman Bin Faisal University (IRB-2021-01-453). Written permission from the MoE was obtained before starting the data collection process. Individual written informed consent was a prerequisite for data collection. All questionnaires were anonymous and the collected data were kept private and used only for the purposes of this study. To assess the acceptability and applicability of the methods, a pilot study was conducted with 20 teachers who were excluded from the sample.

Data were collected by senior medical students trained and supervised by two occupational physicians. Three elements of the questionnaire, namely sociodemographic and health-related questions, the Standardized Nordic Questionnaire, and the Oswestry Low Back Pain Disability Questionnaire, were included in the training (21–23). The training also included anthropometric data (height and weight of the participants). The questionnaires were reverse-translated to ensure maximum quality and no misunderstanding.

### 2.2 Questionnaires

#### 2.2.1 Sociodemographic, health and work-related questions

This section included questions on sociodemographic variables such as age, sex, and marital status. Other questions included health-related lifestyle habits such as smoking, perceived levels of physical activity, and perceived stress. Work-related questions included questions on teaching years, number of classes per week, type of school, standing status during teaching, and the subjects taught (7, 21, 24).

#### 2.2.2 Standardized Nordic Questionnaire

This study used valid and reliable low back pain screening and analysis questions from the Standardized Nordic Questionnaire. The focus was primarily on the symptom duration of low back trouble (ache, pain, or discomfort) in the past, including the last 7 days, the past year, and the past lifetime. A response of “yes” to any question was coded as a “yes,” whereas a “no” response to all questions was coded as a “no” (21).

#### 2.2.3 Oswestry Low Back Pain Disability Questionnaire

The Oswestry Low Back Pain Disability Questionnaire is a valid and reliable tool for assessing persistent functional impairment caused to low back pain. It consists of 10 sections including six statements on how back pain affects each of the following areas: pain severity, lifting, personal care, walking, sitting, sex life, standing, social life, sleeping, and travel. The total score was computed and converted to a percentage that could then be interpreted based on pre-specified cut-off points as follows: 0–20% minimal disability, 20.1–40% moderate disability, 40.1–60% severe disability, and 60.1–80% crippled. Participants with scores >80% were considered bedridden or had subconsciously exaggerated their symptoms (23).

#### 2.2.4 Anthropometric data

The participants’ anthropometric data, including body height (m) and weight (kg), were measured at the time of data collection using four digital scales, with their consent. The Medical Digital Column Scales by Charder Electronic Co, model MS4900 and MS4970, were used. The scales were calibrated to ensure high-quality measurements. Using these measurements, participants were then classified to categories of body mass index (BMI) as follows; underweight less than 18.5 kg/m^2^, normal weight is 18.5–24.9 kg/m^2^, overweight is 25–29.9 kg/m^2^, and obese if BMI was above 30 kg/m^2^ (25).

#### 2.2.5 Statistical analysis

The main outcome was the presence of low back pain during the past 12 months, and the secondary outcome was the disability in teachers who had low back pain. Disability was computed by summing all scores and categorizing them according to the questionnaire criteria. Continuous variables are described as means ± standard deviation and categorical variables as frequencies and percentages. Chi-square and Fisher’s exact tests were used to assess bivariate associations between categorical variables, whereas t-tests and analysis of variance (ANOVA) were used to assess associations with continuous variables. *Post hoc* analysis was used for significant associations with the Sidak correction due to assumptions of independence. The choice of inclusion of variables into the regression model was based on Directed Acyclic Graph of relationships between explanatory variables and the outcomes. Unadjusted and adjusted binary logistic regression analyses were performed to compute the odds ratios (ORs) for the odds of low back pain and their accompanying 95% confidence intervals (CIs). The level of significance of 5% was considered appropriate. Model fit diagnostics were performed to ensure a good model fit, and the model than minimized both the AIC and BIC was used. Stata statistical software version 15 was used for all analyses.

## 3 Results

### 3.1 Sociodemographic, health and teaching related characteristics

A total of 601 teachers participated in the study. The overall mean age was 40.31 ± 8.13 years. The male-to-female ratio was similar. Approximately 82% of the total sample were married, whereas only 1.33% were widowed. Regarding obesity, 38.9% were overweight and 33.9% were obese. Chronic conditions were present in 25.8% of all teachers, and 3.16% were current smokers. Only 38.4% of the participants reported exercising regularly. Examining teaching characteristics, most participants taught science subjects (23.96%), followed by language and humanities (22.8 and 22.63%, respectively). Over half of the teachers taught between 11 and 20 classes per week, and the majority belonged to private schools. Only 16.3% of participating teachers had additional jobs (Table 1).

**Table 1:** Sociodemographic, health and teaching related characteristics of study sample.

Characteristic	*N* (%) 601 (100.00)
Age (μ, SD)	40.31 (08.13)
Gender	
Males	297 (49.42)
Females	304 (50.58)
Marital status	
Single	75 (12.48)
Married	495 (82.36)
Divorced	23 (03.83)
Widowed	8 (01.33)
Body Mass Index (kg/m^2^)	
Underweight	14 (02.33)
Normal weight	149 (24.79)
Overweight	234 (38.94)
Obese	204 (33.94)
Chronic conditions	
No	446 (74.21)
Yes	155 (25.79)
Perceived stress	
Never	122 (20.30)
Sometimes	308 (51.25)
Often	91 (15.14)
Always	80 (13.31)
Smoking status	
Non-smoker	524 (87.19)
Ex-smoker	58 (09.65)
Current smoker	19 (03.16)
Regular exercise	
No	370 (61.56)
Yes	231 (38.44)
Teaching area	
Languages	137 (22.80)
Humanities and social sciences	136 (22.63)
Sciences	144 (23.96)
Mathematics	79 (13.14)
Computer and applied sciences	66 (10.98)
Others	39 (06.49)
Number of classes per week	
≤ 10 classes	80 (13.31)
11 ≤ 20 classes	320 (53.24)
> 21 classes	201 (33.44)
Type of school	
Private	396 (66.67)
Governmental	198 (33.33)
Work an extra job	
No	503 (83.69)
Yes	98 (16.31)
Had low back trouble?	
No	225 (37.44)
Yes	376 (62.56)

### 3.2 Low back pain symptoms

Table 2 describes the presence and symptoms of low back pain according to the Nordic questionnaire. Of the total number of teachers, 62.56% reported low back pain. Of those, 25.5% had been previously hospitalized due to that pain and 7.7% had to change the nature of their jobs. Among those with back pain, 16.49% reported that they experienced pain on a daily basis during the past 12 months. Back pain had reduced work activity in 63.56% of complainers, whereas leisure activity was reportedly reduced in 62.9% of complainers. Additionally, 12.5% of those with back pain reported that it had prevented them from usual work for more than 30 days during the past 12 months. Over 38% had visited a doctor due to the pain, and 51.6% reported that the pain had occurred within the 7 days preceding their participation in this study.

**Table 2:** Low back pain symptoms among participating teachers.

Standardized Nordic questions	*N* (%) 376 (100.00)
Had been hospitalized?	
No	280 (74.47)
Yes	96 (25.53)
Had to change job?	
No	347 (92.29)
Yes	29 (07.71)
Total length of time of low back pain in the past 12 months?	
0 days	21 (05.59)
1–7 days	130 (34.57)
8–30 days	69 (18.35)
More than 30 days, but not everyday	94 (25.00)
Everyday	62 (16.49)
Reduced work activity in the past 12 months?	
No	137 (36.44)
Yes	239 (63.56)
Reduced leisure activity in the past 12 months?	
No	138 (37.10)
Yes	234 (62.90)
Total length of time of low back pain prevented usual work in the past 12 months?	
0 days	92 (24.47)
1–7 days	179 (47.61)
8–30 days	58 (15.43)
More than 30 days	47 (12.50)
Seen a doctor in the past 12 months?	
No	232 (61.70)
Yes	144 (38.30)
Had low back pain during the last 7 days?	
No	182 (48.40)
Yes	194 (51.60)

### 3.3 Associations between low back pain, sociodemographic, health and teaching characteristic

The results of the bivariate analyses have showed that a highly statistically significant difference was observed between age and presence of low back pain (*p* < 0.001). Back pain was also significantly higher among females than males (73.36 and 51.52%, respectively). Marital status was associated with back pain; participants who were currently married or had a history of marriage tended to report low back pain more often than single participants (*p* = 0.001). There was also a statistically significant difference in chronic conditions and regular exercise, where participants who had reported a chronic condition and those who did not exercise reported low back pain more than their counterparts (70.32 and 65.95%, respectively) (*p*- value = 0.02 and *p*- value = 0.03 respectively). For perceived stress, a highly significant difference was observed, which reflected an increase in the reporting of back pain with increasing stress levels (*p*-value <0.001). No statistically significant difference were observed between the BMI and smoking status (Table 3).

**Table 3:** Associations between self-reported low back pain and sociodemographic, health and teaching characteristics.

Characteristics	Presence of low back pain		*p*-value
	Absent 225 (37.44)	Present 376 (62.56)	
Age (μ, SD)	38.4 (08.4)	41.5 (07.7)	< 0.001^*^
Gender			< 0.001^**^
Males	144 (48.48)	153 (51.52)	
Females	81 (26.64)	223 (73.36)	
Marital status			0.001^***^
Single	41 (54.67)	34 (45.33)	
Married	176 (35.56)	319 (64.44)	
Divorced	8 (34.78)	15 (65.22)	
Widowed	0	8 (100.00)	
Body Mass Index (kg/m^2^)			0.37^**^
Underweight	6 (42.86)	8 (57.14)	
Normal weight	64 (42.95)	85 (57.05)	
Overweight	81 (34.62)	153 (65.38)	
Obese	74 (36.27)	130 (63.73)	
Chronic conditions			0.02^**^
No	179 (40.13)	267 (59.87)	
Yes	46 (29.68)	109 (70.32)	
Perceived stress			< 0.001^**^
Never	62 (50.82)	60 (49.18)	
Sometimes	122 (39.61)	186 (60.39)	
Often	23 (25.27)	68 (74.73)	
Always	18 (22.50)	62 (77.50)	
Smoking status			0.21^**^
Non-smoker	190 (36.26)	334 (63.74)	
Ex-smoker	25 (43.10)	33 (56.90)	
Current smoker	10 (52.63)	9 (47.37)	
Regular exercise			0.03^**^
No	126 (34.05)	244 (65.95)	
Yes	99 (42.86)	132 (57.14)	
Teaching area			0.21^**^
Languages	46 (33.58)	91 (66.42)	
Humanities and social sciences	45 (33.09)	91 (66.91)	
Sciences	54 (37.50)	90 (62.50)	
Mathematics	37 (46.84)	42 (53.16)	
Computer and applied sciences	30 (45.45)	36 (54.55)	
Others	13 (33.33)	26 (66.67)	
Number of classes per week			0.03^**^
≤ 10 classes	40 (50.00)	40 (50.00)	
11 ≤ 20 classes	118 (36.88)	202 (63.12)	
> 21 classes	67 (33.33)	134 (66.67)	
Type of school			0.004^**^
Private	132 (33.33)	264 (66.67)	
Governmental	90 (45.45)	108 (54.55)	
Work an extra job			0.94^**^
No	188 (37.33)	315 (62.62)	
Yes	37 (37.76)	61 (62.24)	

*p-value obtained from a *T*-test.

**p-value obtained from Chi-squared tests.

***p-values obtained from Fisher’s exact test.

### 3.4 Associations between disability due to low back pain, sociodemographic, health and teaching characteristics

Table 4 presents a subgroup analysis of teachers who reported low back pain and level of disability according to the Oswestry Low Back Pain Disability Questionnaire. Among the 376 teachers who reported low back pain, there was minimal disability (64.63%), moderate disability (29.79%), and severe disability (4.79%); only three (0.8%) were considered crippled. Age was highly associated with the disability score, and an increase in age was observed with an increase in severity (*p* = 0.002) (Significant *p* for trend). A statistically significant difference in the disability score was observed with sex, where females were seen more frequently in the moderate and crippled categories (*p* = 0.005). In addition, the number of teachers in private schools was higher than their counterparts in the moderate, severe, and crippled disability categories (34.09, 5.68, and 1.14%, respectively) (*p* = 0.006). No other significant associations were observed.

**Table 4:** Subgroup analyses of disability among teachers with self-reported low back pain.

Characteristic	Minimal 0–20% 243 (64.63)	Moderate 21–40% 112 (29.79)	Severe 41–60% 18 (4.79)	Crippled 61–80% 3 (0.8)	*p*-value
Age (μ, SD) ^‡^	40 (8.0)	43 (7.0)	45 (6.0)	45 (1.0)	0.002^*^
Gender					0.005^**^
Males	114 (74.51)	30 (19.61)	8 (05.23)	1 (00.65)	
Females	129 (57.85)	82 (36.77)	10 (4.48)	2 (00.9)	
Marital status					0.64^**^
Single	23 (67.65)	11 (32.35)	0	0	
Married	208 (65.2)	93 (29.15)	15 (04.7)	3 (0.94)	
Divorced	7 (46.67)	6 (40.0)	2 (13.33)	0	
Widowed	5 (62.5)	2 (25.0)	1 (12.5)	0	
Body Mass Index (kg/m^2^)					0.49^**^
Underweight	7 (87.5)	1 (12.5)	0	0	
Normal weight	51 (60.0)	31 (36.47)	2 (2.35)	1 (1.18)	
Overweight	102 (66.67)	39 (25.49)	10 (6.54)	2 (1.31)	
Obese	83 (63.85)	41 (31.54)	6 (4.62)	0	
Chronic conditions					0.07^**^
No	182 (68.16)	71 (26.59)	11 (4.12)	3 (1.12)	
Yes	61 (55.96)	41 (37.61)	7 (6.42)	0	
Perceived stress					0.20^**^
Never	48 (80.00)	11 (18.33)	1 (1.67)	0	
Sometimes	123 (66.13)	53 (28.49)	9 (4.84)	1 (0.54)	
Often	38 (55.88)	25 (36.76)	4 (5.88)	1 (1.47)	
Always	34 (54.84)	23 (37.1)	4 (6.45)	1 (1.61)	
Smoking status					0.68^**^
Non-smoker	213 (63.77)	102 (30.54)	16 (4.79)	3 (0.90)	
Ex-smoker	23 (69.7)	9 (27.27)	1 (3.03)	0	
Current smoker	7 (77.78)	1 (11.11)	1 (11.11)	0	
Regular exercise					0.17^**^
No	155 (63.52)	79 (32.38)	9 (3.69)	1 (0.41)	
Yes	88 (66.67)	33 (25.0)	9 (06.82)	2 (1.52)	
Teaching area					0.95^**^
Languages	58 (63.74)	27 (29.67)	5 (5.49)	1 (1.1)	
Humanities and social sciences	61 (67.03)	23 (25.27)	5 (5.49)	2 (2.2)	
Sciences	56 (62.22)	30 (33.33)	4 (4.44)	0	
Mathematics	24 (57.14)	15 (35.71)	3 (7.14)	0	
Computer and applied sciences	25 (69.44)	10 (27.78)	1 (2.78)	0	
Others	19 (73.08)	7 (26.92)	0	0	
Number of classes per week					0.21^**^
≤ 10 classes	30 (75.0)	9 (22.5)	1 (2.50)	0	
11 ≤ 20 classes	130 (64.36)	63 (31.19)	6 (2.97)	3 (1.49)	
> 21 classes	83 (61.94)	40 (29.85)	11 (08.21)	0	
Type of school					
Private	156 (59.09)	90 (34.09)	15 (5.68)	3 (1.14)	0.006^**^
Governmental	84 (77.78)	21 (19.44)	3 (2.78)	0	
Work an extra job					0.21^**^
No	202 (64.13)	98 (31.11)	13 (4.13)	2 (0.63)	
Yes	41 (67.21)	14 (22.95)	5 (8.2)	1 (1.64)	

^‡^Significant *p* for trend (< 0.001).

*p-value obtained from an ANOVA – *Post hoc* analysis shows a significant difference between minimal and moderate disability (*p*-value = 0.03).

**p-value obtained from Fisher’s exact test.

**Figure 1:**
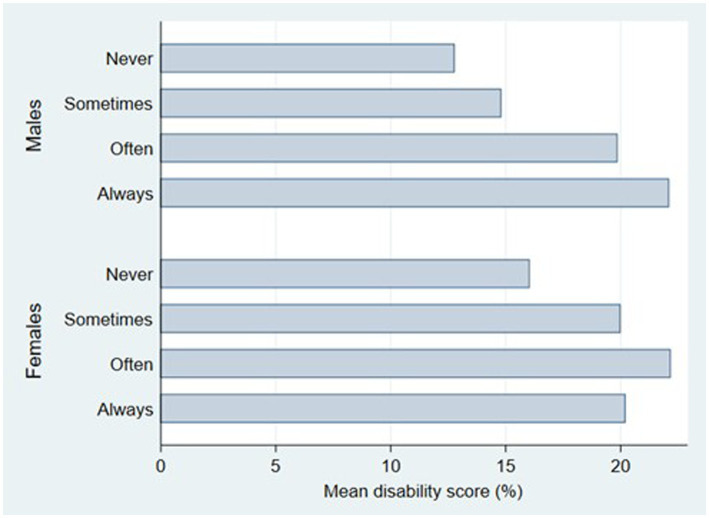
Mean score of disability for males and females according to perceived stress.

Figure 1 shows the mean disability scores for males and females according to the level of perceived stress. In males, a clear and gradual increase in the mean disability score was observed with increasing levels of perceived stress. A general increase was also observed in women.

### 3.5 Multivariable associations and odds of low back pain

In the simple logistic regression model, age, sex, perceived regular exercise, number of classes and perceived stress were statistically significant. The multiple logistic regression has found that age was a significant predictor of low back pain after adjusting for all other variables (adjusted OR = 1.04, 95% CI = 1.02–1.07). Similarly, sex was a significant predictor; a more than 2-fold increase in odds for females was observed compared to males (adjusted OR 2.11, 95% CI = 1.45–3.05). The number of classes given by participating teachers per week was also a significant predictor, teachers who had 10 to 20 classes per week was higher than that of teachers who had less than 10 classes per week (adjusted OR = 1.77, 95% CI = 1.05–3.00). Moreover, the odds were even greater for teachers who had over 21 classes per week when compared to teachers who had less than 10 classes (adjusted OR = 2.09, 95% CI = 1.19–3.68). A significantly increased odds was observed for teachers who reported stress compared to those who had never reported stress (adjusted OR = 2.1, 95% CI = 1.12–3.93), the odds was similar for those who had perceived stress all the time (95% CI = 2.07, 95% CI = 1.05–4.07) (Table 5).

**Table 5:** Unadjusted and adjusted binary logistic regression analysis.

Predictors	Unadjusted model			Adjusted model		
	Unadjusted OR	95% CI	*p*-value	OR	95% CI	*p*-value
Age	1.04	1.02–1.07	< 0.001	1.04	1.02–1.07	< 0.001
Sex						
Males	Ref			Ref		
Females	2.59	1.84–3.64	< 0.001	2.11	1.45–3.05	< 0.001
Exercise						
No	1.45	1.03–2.03	0.03	1.32	0.92–1.89	0.12
Yes	Ref			Ref		
Number of classes per week						
≤ 10 classes	Ref			Ref		
11 ≤ 20 classes	1.71	1.04–2.80	0.03	1.77	1.05–3.00	0.03
> 21 classes	2.00	1.18–3.38	0.01	2.09	1.19–3.68	0.01
Perceived stress						
Never	Ref			Ref		
Sometimes	1.57	1.03–2.40	0.03	1.22	0.78–1.92	0.37
Often	3.05	1.69–5.51	< 0.001	2.10	1.12–3.93	0.02
All the time	3.55	1.88–6.70	0.001	2.07	1.05–4.07	0.03

## 4 Discussion

In this study, the presence of LBP and its risk factors in the previous 12 months was investigated as a primary outcome among teachers of both sexes at secondary schools in Saudi Arabia’s eastern region, and as a secondary outcome to determine the score of disability among those who had reported low back pain.

Analysis of the data obtained from the surveys showed that 62.56% of the participating teachers had experienced lower back pain at least once in the last 12 months. These results with high prevalence agree with those obtained by Raizah et al. and Abdulmonem et al. (8, 13, 14, 26, 27). The study by Darwish et al. (13), conducted among the secondary school female teachers in the eastern province, concluded that 63.8% of them reported that the lower back was the most common site of pain they experienced, which is very close to the 62.56% reported in our study. The results in the same culture suggest that a few specific factors, such as teaching classes, long work hours, and high levels of perceived stress at work, may have significant effects (17).

However, this result differs from those of certain studies, such as a longitudinal study on secondary school teachers in Malaysia, which found a prevalence of 44% (28). The differences could be explained by the type of study and differences in ethnicity.

According to a national study on employees in the US, 10.7% of those with regular and severe LBP and 6.1% of those with any LBP stopped working, changed jobs, or significantly modified their work activities in the previous 3 months as a result of their LBP (29).

In the current study, back pain decreased work activity in 63.56% of complainers, while leisure activity was reduced in 62.9%, adding to the fact that 7.7% of those who complained had to change the nature of their jobs. These findings show a significant burden and negative impact of back pain among teachers, which might impair work performance, and are corroborated by a World Health Organization report. Low back pain is the primary cause of the total burden of musculoskeletal diseases and is responsible for 7.4% of all years lived with disability (YLDs) worldwide (30).

The results of the present study showed, the difference between men and women, with women reporting LBP more frequently than men. These results are consistent with those reported by Althomali et al. and Erick et al. However, other studies did not reveal any significant differences between men and women (6, 7, 14, 28, 31, 32). Notably, this trend of a higher prevalence of lower in women is present worldwide, as confirmed by a systematic review published in 2011 (32). It has been suggested that women may report pain at a higher rate than men due to factors such as lower physical strength, a longer work time, or that male teachers exercised more frequently than female teachers (7, 14, 32).

According to the results of the current study, age has been found to be a significant predictor of low back pain. This result is in line with that of Erick et al., who also found a significant correlation between low back pain and aging. The possibility that they spent more years teaching as they grew older could help to explain this result. People’s muscle mass steadily declines with age, their connective tissue loses its elasticity, and the cartilage between their joints thins (7, 33).

Some studies have linked irregular exercise to low back pain (5, 16, 26). In fact, after performing a logistic regression, our study did not clearly demonstrate any association between irregular exercise to low back pain which correlates with the results of the study by Zamri and Yue (6, 28). This indicates that to obtain more informative findings, we need to ask more focused questions on the types and lengths of exercises.

As for occupation-related risk factors, the stressful nature of the occupation had an undeniable adverse impact on the health of the participants, making them more prone to LBP and more exposed to occupational stressors, as statistically proven in our study as well as in a study published by Bogaert et al. (34) supporting this mind–body connection, highlighting the importance of teachers’ mental health and their reflection on their physical state of health, many of which may not have been realized. This was exactly what we found in our study, with the notable correlation between teachers’ back pain and perceived stress being one of the most significant findings. Low back pain becomes more prevalent and disabling as stress levels increase. These findings were consistent with those of previous studies (7, 8, 27, 28).

Several factors may have influenced the teachers’ stress. Increased workload, more classes dealing with young adolescents’ emotional and behavioral issues, and loss of social support are just a few of the challenges teachers face (28, 35). Therefore, it is important to identify potential workplace issues that contribute to teachers’ stress. Specialized stress management programs may help reduce low back pain and disability. We also recommend conducting more research to determine the association between mental illnesses and low back pain in teachers using reliable assessments like PHQ-9 (Patient Health Questionnaire-9) and GAD-7 (General Anxiety Disorder-7) scales, considering the highly significant relationship between perceived stress and low back pain.

Similarly, physical stress, represented in the occupational context of being a teacher by the number of classes taught per week, could be a risk factor for LBP among secondary school teachers, in addition to the nature of the class itself. As predicted, our study found that the greater the number of classes, the greater the LBP risk of low back pain. This study largely confirms the findings of earlier research in this field relating the number of classes to the incidence of low back pain, whether in national or worldwide studies (4, 6, 13, 14, 28, 35). This result might be explained by the prolonged standing or sitting during teaching classes, which could be considered an aggravation factor; while dealing with students during class, teachers often require physical effort or the maintenance of particular postures for an extended period of time just to be on the same level as those students, such as kneeling or bending down when interacting with them (18, 31).

Surprisingly, BMI was not positively correlated with presence of LBP in the current study (34). However, the results of this study disagree with those of other studies (7, 8, 14, 27, 29, 31). Despite many previous studies demonstrating the positive association between BMI and lower back pain through different mechanisms, the insignificant relationship in our study could suggest that the obesity incidence in our study population manifested late and therefore the obesity impact on the lower back will be developed later (36).

LBP is one of the main factors contributing to lower quality of life, work loss, and participation restrictions worldwide (33). Work-related LBP due to work affects everyone, including teachers. Therefore, the Oswestry Disability Index was used to assess how this illness impairs a person’s capacity for daily tasks. Most Polish teachers who complained in the Rottermund et al. study (86–87%) had only mild impairment, with no significant sex differences. In our study, 64.63% of low back pain sufferers reported minimal disability, which is consistent with the data observed in the study by Eric et al. (7). In contrast to what was seen in Polish teachers, our study had a larger percentage of moderate disabilities (29.79%), with a statistically significantly higher percentage of females. This research revealed sex differences comparable to those reported by Ya et al. in their study of Chinese office workers. High stress levels and the higher prevalence of pain in females may be one of the primary reasons for them having a higher disability levels. For further illustration, Figure 1 demonstrates the positive association between stress and disability levels (4, 37).

The most important finding from our data is the substantial link between age and disability score. This is consistent with a WHO report. It is crucial to increase health awareness and implement preventative measures, such as self-care and ergonomic changes, among teachers (33).

### 4.1 Limitation

This study has several limitations that must be noted. Four types of electronic weight scales were used to collect the data, which may have led to slightly varied weight readings and, as a result, slightly variable BMI calculations. Furthermore, this study did not consider the stated pregnancy status of the female participants, which could have been a confounding factor if the pregnant participants had lower back discomfort. Participants were also questioned about their symptoms from the previous year, which required them to recall, and may have allowed for recall bias. An additional limitation, the teachers under our study were in the two spectrums of 22 to 62 (low to high age). This could confound our results because did not differentiate between different ages. Also, we did not check the teacher standing hours in our study, which can be effective, because it is difficult to know the precisely standing hours. The use of percieved levels of excercise and percieved stress may have added a level of subjectivity to the results. The study may have benefited more from validated tools for these variables, however this would have greatly increased the number of questions.

### 4.2 Conclusion

The present study has shown that low back pain is highly prevalent among secondary school teachers in the eastern region of Saudi Arabia, along with having a significant disability index which apparently affects the quality of their lives. The presence of LBP and disability were positively correlated with female sex, advancing age, perceived stress, and the number of classes per week. On other hand, low back pain and disability were not associated with smoking or increased BMI. The variety of LBP risk factors among teachers emphasize the need for implementing different preventative measures, such as promotional campaigns aiming to educate teachers about workplace ergonomics and how to deal with physical and emotional stress, as well as educating decision-makers to reconsider the weekly number of classes assigned to a single teacher and to set a maximum reasonable number of classes per week. Furthermore, early-detection programs aimed at screening for common disorders in a certain work environment are recommended. For example, screening for psychological disorders and lower back pain in teachers.

## Data availability statement

The original contributions presented in the study are included in the article/supplementary material, further inquiries can be directed to the corresponding author.

## Ethics statement

The studies involving humans were approved by the Institutional Review Board of the Imam Abdulrahman Bin Faisal University (IRB-2021-01-453). The studies were conducted in accordance with the local legislation and institutional requirements. The participants provided their written informed consent to participate in this study.

## Author contributions

AA: Conceptualization, Methodology, Supervision, Visualization, Writing – original draft, Writing – review & editing. DA: Data curation, Investigation, Writing – original draft. TA: Data curation, Investigation, Project administration, Writing – original draft. MK: Conceptualization, Investigation, Project administration, Writing – original draft. MA: Data curation, Investigation, Project administration, Writing – original draft. RaA: Data curation, Investigation, Project administration, Writing – original draft. KA: Visualization, Writing – original draft, Writing – review & editing. HA: Conceptualization, Supervision, Visualization, Writing – review & editing. ReA: Conceptualization, Formal analysis, Methodology, Software, Visualization, Writing – original draft, Writing – review & editing.
